# Changes in EEG Brain Connectivity Caused by Short-Term BCI Neurofeedback-Rehabilitation Training: A Case Study

**DOI:** 10.3389/fnhum.2021.627100

**Published:** 2021-06-24

**Authors:** Youhao Wang, Jingjing Luo, Yuzhu Guo, Qiang Du, Qiying Cheng, Hongbo Wang

**Affiliations:** ^1^Academy for Engineering and Technology, Fudan University (FAET), Shanghai, China; ^2^Jihua Laboratory, Foshan, China; ^3^School of Automation Science and Electrical Engineering, Beihang University, Beijing, China

**Keywords:** brain-computer interface, electroencephalogram, motor imagery, neurofeedback-rehabilitation, short-term training, event-related potentials, brain-network analysis

## Abstract

**Background:**

In combined with neurofeedback, Motor Imagery (MI) based Brain-Computer Interface (BCI) has been an effective long-term treatment therapy for motor dysfunction caused by neurological injury in the brain (e.g., post-stroke hemiplegia). However, individual neurological differences have led to variability in the single sessions of rehabilitation training. Research on the impact of short training sessions on brain functioning patterns can help evaluate and standardize the short duration of rehabilitation training. In this paper, we use the electroencephalogram (EEG) signals to explore the brain patterns’ changes after a short-term rehabilitation training.

**Materials and Methods:**

Using an EEG-BCI system, we analyzed the changes in short-term (about 1-h) MI training data with and without visual feedback, respectively. We first examined the EEG signal’s Mu band power’s attenuation caused by Event-Related Desynchronization (ERD). Then we use the EEG’s Event-Related Potentials (ERP) features to construct brain networks and evaluate the training from multiple perspectives: small-scale based on single nodes, medium-scale based on hemispheres, and large-scale based on all-brain.

**Results:**

Results showed no significant difference in the ERD power attenuation estimation in both groups. But the neurofeedback group’s ERP brain network parameters had substantial changes and trend properties compared to the group without feedback. The neurofeedback group’s Mu band power’s attenuation increased but not significantly (fitting line slope = 0.2, *t*-test value *p* > 0.05) after the short-term MI training, while the non-feedback group occurred an insignificant decrease (fitting line slope = −0.4, *t*-test value *p* > 0.05). In the ERP-based brain network analysis, the neurofeedback group’s network parameters were attenuated in all scales significantly (*t*-test value: *p* < 0.01); while the non-feedback group’s most network parameters didn’t change significantly (*t*-test value: *p* > 0.05).

**Conclusion:**

The MI-BCI training’s short-term effects does not show up in the ERD analysis significantly but can be detected by ERP-based network analysis significantly. Results inspire the efficient evaluation of short-term rehabilitation training and provide a useful reference for subsequent studies.

## Introduction

Electroencephalograph (EEG)-based BCI systems is often applied in combination with motor imagery (MI) paradigm (Alkadhi et al., 2005) for neurorehabilitation training (Kumar et al., 2016; Baig et al., 2017; Oikonomou et al., 2017; Cheng et al., 2018), especially for enhancing motor recovery from brain injuries such as stroke hemiplegia (Buch et al., 2008; Zimmermann-Schlatter et al., 2008; Daly et al., 2009; Langhorne et al., 2009; Barclay et al., 2020). Neurofeedback (NF) is also commonly applied in the BCI system. Thus cortical movement intention can be transferred to physical activity or stimulation that feeds back to the patient as a consequent response, forming a closed-loop neural circuit (Yu et al., 2015; Zich et al., 2015; Sitaram et al., 2017). Clinical studies have shown improvement in neurorehabilitation using MI-BCI system with NF (Prasad et al., 2009; Caria et al., 2011; Shindo et al., 2011; Ramos-Murguialday et al., 2013; Mukaino et al., 2014), and results are supported by the underlying mechanisms of neural plasticity and brain reorganization (Rozelle and Budzynski, 1995; Ang et al., 2014).

Neurorehabilitation assessment is essential for both patients as well as BCI system evaluation. Clinical assessments of physical function restoration such as functional upper extremity test (FMA), wolf motor function test (WMFT) are used as typical methods (Rozelle and Budzynski, 1995; Mihara et al., 2013; Ang et al., 2014; Li et al., 2014; Kim et al., 2016; Leeb et al., 2016). However, most physical assessments are only applicable after substantial functional recovery with a long training period and are the indirect measure of brain injury recovery. Researchers have been studying brain imaging techniques such as functional Magnetic Resonance Imaging (fMRI) (Song et al., 2014; Young et al., 2014), EEG (Daly and Wolpaw, 2008; Ono et al., 2015), and electromyogram (EMG) (Rozelle and Budzynski, 1995; Daly and Wolpaw, 2008). The goal is to find new assessment methods to analyze the brain directly and observe subtle changes in neural reorganization. For BCI rehabilitation, the challenge is to establish an EEG quantitative standard to evaluate the rehabilitation effect. MI as a typical BCI rehabilitation paradigm varies in its performance when applying different feedback strategies (Ahn and Jun, 2015; Marzbani et al., 2016; Renton et al., 2017). There are other factors such as induction paradigm or training engagement, may affect potential brain recovery, thus make it more important to find direct and rapid measurements for BCI rehabilitation using EEG.

For BCI EEG analysis, sensorimotor rhythm (SMR) of neurophysiological oscillations and event-related potentials (ERPs) are commonly used as neurophysiological features. As a particular example of SMR, desynchronization/synchronization (ERD/ERS) modulation during MI or movement execution (Pfurtscheller and Da Silva, 1999; Graimann et al., 2009; Nicolas-Alonso and Gomez-Gil, 2012) is proportional to the motor function’s impaired level of patients (Matsumoto et al., 2010; Rossiter et al., 2014; Naros and Gharabaghi, 2015; Soekadar et al., 2015). And it was found to be improved in the prolonged MI-BCI rehabilitation (Rozelle and Budzynski, 1995; Pfurtscheller and Da Silva, 1999; Yoshida et al., 2016). The ERPs as EEG averages are direct amplitude changes in response to exhibited events (Kok, 1997). Both signals characterize as potential recovery measures, given that they may carry information about underlying mechanisms of brain recovery. What’s more, the functional connectivity of brain networks is another strategy to reveal changes in neural activity. For example, brain network analysis based on fMRI has been used in clinical-pathological studies (Van Den Heuvel and Pol, 2010). Compared to the fMRI, the convenience and high temporal resolution of the EEG signal has led to an increasing number of scholars using it to analyze the brain networks (Varela et al., 2001; Wang et al., 2010; Faith et al., 2011; Sakkalis, 2011; Carter et al., 2012; Stam and Van Straaten, 2012; Belardinelli et al., 2017). Further studies use the EEG to apply graph theory on the cortical network (Bullmore and Sporns, 2009; Fallani et al., 2013; Cheng et al., 2015) to measure brain changes by rehabilitation training (Brown, 1970; de Vico Fallani et al., 2014; Philips et al., 2017).

Studies mentioned above show that neural functional changes reflected by EEG signals are reliably correlated with changes in physical function. Still, the results are observed only after prolonged training, which may not be comprehensive enough. Thus, we consider the short-term effects of BCI on brain activity. BCI training with feedback could alternately enhance and suppress spontaneous rhythmic activity for short periods (Nowlis and Kamiya, 1970; Beatty et al., 1974; Sterman, 1974) and leads to sustained changes in neural activity (Kaplan, 1975; Wyler et al., 1976). Lin et al. found that short-term training leading to significant neural activity changes in brain network by using functional connectivity of fMRI (Lin et al., 2017). In neurorehabilitation, Tsuchimoto et al. (2019) found that BCI training with neurofeedback can effect on patients’ EEG synchrony in the short term. We can infer that the short-term MI-BCI rehabilitation training variations based on EEG signals may also have the ability to interpret the rehabilitation process. Evaluating those variations can help to portray the recovery process more accurately. Yet, the variations are still unclear, and an efficient and rapid recovery assessment method of short-term MI-BCI rehabilitation training has not been proposed. Using the EEG to study the state of neural signal expression in a short time may provide a new approach to measuring the effects of rehabilitation training.

Our study investigated how the short-term MI-BCI training affects the human brain and uses EEG signals to evaluate it. We used EEG’s Mu band power attenuation to analyze the impact of short-term rehabilitation training and use network methods to analyze the effectiveness of exercise on various network scales. In section II, the experimental data are presented, and the analysis methods are described. Section III presents the experimental results of the short-term ERD modulation and the ERP-based cortical network, respectively. Discussion and conclusions are presented at the end.

## Materials and Methods

### Data Acquisition

We used left- and right-handed motor imagery data from a publicly available dataset (Kaya et al., 2018). All 5 subjects underwent 3 days of MI-BCI training were selected, of which four subjects with no visual feedback and one subject with visual feedback. In all experiments, an EEG distribution with 19 electrodes in the International Standard 10–20 system was used. Data was acquired using a medical-grade EEG-1200 recording system with a JE-921A acquisition cassette (Nihon Kohden, Japan) and band-filtered at 0.53∼70 Hz at the recording phase. Participants were seated in a chair and observed a computer screen about 200 cm in front of the BCI system. A typical rehabilitation training of left/right hand MI was applied as the experiment paradigm. Two formats of experiments were conducted, a “non-feedback” mode as well as a “feedback” mode, introduced as follows.

#### Non-feedback Data

**FIGURE 1:**
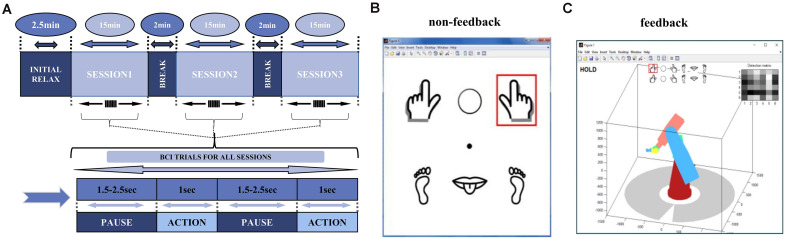
Experimental Paradigm. **(A)** The experimental paradigm of the data is divided into three 15-min sessions, each containing 300 BCI trials, with an average duration of 3 s per trial, including about 2 s of pause and 1 s of the action. **(B)** Icons seen by the subjects during the non-feedback experiment. Subjects follow the instructions in the red box for the MI task. **(C)** Computer instructions for Feedback experiment. Subject are able to move the robotic-arms as feedback in the MI-task.

The whole process lasted 51.5 min, assembled from three 15-min sessions, with a 2.5-min break to initialize the system before the session start, followed by a 2-min break between the two sessions for the subject to relax (Figure 1C). Each session contained 300 trials in total, each consisting of pause and action phases. The pause phase had a duration of 1.5–2.5 s randomly, with an average of 2 s. During the action phase, the screen showed a GUI interface with a red square, to instruct the participant to perform the corresponding task for 1 s (Figure 1A). The red square upon the left- or right-handed cartoon image indicated the grasping MI task, and upon the middle circle indicated a “hold” task with no imagery (Figure 1A). The experiment was carried out on 3 days at irregular intervals. The four subjects of non-feedback paradigm were labeled as A, C, D, E in this article.

#### Feedback Data

The feedback paradigm had the same overall steps as the non-feedback paradigm, however subjects were asked to control actions of a 3D virtual robotic arm. During the action phase, the screen showed a virtual robotic-arm bellow the task icons. Depending on the real-time decoding analysis of Mu-suppression, robotic arm appeared to move left/right or stay “hold” (Figure 1B). The robotic-arm moved as the feedback of an imagery success. The first session followed the same steps as in the non-feedback paradigm. In second and third sessions, subject’s imagery was actively performed, and the movement of the virtual robotic arm was determined by subjects themselves initially. It was then set as specific task sequences, e.g., to “move two units to the left” or to “move 1 unit to the left and then three units to the right.” We labeled the feedback subject as subject B later in the article. We arranged EEG data of 3 days in parallel for statistical analysis for each subject. In each day, left- or right-hand imagery task trials were used with all “hold” trials removed for EEG analysis in this article.

### Mu Suppression Score

ERD/ERS in MI task is calculated by the power spectral density(PSD) of EEG signals in the personalized frequency range, typically at 8–13 Hz known as the Mu band (Kuhlman, 1978; Pfurtscheller and Da Silva, 1999). In the ERD phenomenon, the corresponding region of primary motor cortex (M1) in the hemisphere contralateral to the movement is attenuated. In this study, the C3 and C4 electrode positions from the 10 to 20 international system are located close to the M1 region. Therefore, for the right-hand MI, we used the C3 channel as the contralateral side, and its symmetric electrode C4 as the ipsilateral side. The same applies to the left-hand MI.

The quantification of ERD/ERS can be calculated using the classical approach called Mu-suppression. The obtained EEG signal is first converted to the frequency domain by Fourier transform. Then, we used multi-taper method (Thomson, 1982) to calculate the PSD, selected frequency range with Mu-suppression for individual experiment to derive the band power. Change of contralateral Mu-band energy between the task state and the resting state was evaluated, using the most recent 1-s before task initiation representing resting state (Thomson, 1982; Oberman et al., 2008; Braadbaart et al., 2013). The following formula gives the Mu-suppression score (MuSC):

(1) $$MuSC=-\frac{MuP_{\text{bo}}-MuP_{\text{nbo}}}{MuP_{\text{nbo}}}*100$$

where *MuP*_*bo*_ is the band power of the task state, and *MuP*_*nbo*_ is the band power of the resting state.

As human brain is characterized by inter-individual variability and rapid dynamic changes, we applied a sliding frequency window with a size of 3 Hz (0.67 overlaps) to precisely select the subject-specific Mu-band boundaries. The most suppressed window comparing the MI state (0∼1 s) against the corresponding resting state (−1∼0 s) was chosen as MI-related EEG oscillations for each subject each day. The screening results for subject-specific Mu-band boundaries are presented in Supplementary Table 1.

### Network Analysis

#### Functional Connectivity Estimation

Neuronal oscillations are implicit in the underlying coordination mechanisms of the brain (Singer, 1999; Varela et al., 2001). The channels with EEG signal contain a collection of oscillations of regional neurons. The synchronization of oscillations between channels may indicate that the brain has information flow between regions (Womelsdorf et al., 2007). Functional Connectivity is a method for assessing the synchronization of oscillating signals from channel to channel. The degree between channels indicates how much information is exchanged.

ERPs is any stereotyped electrophysiological response to a stimulus, which have excellent temporal resolution. Considering the immediacy of the short-term changes targeted in this study, we chose ERPs as the basis for brain network calculations. In the scenario of MI, ERPs are generally obtained by trial averaging. Band-pass filtering is commonly used in some EEG studies for data preprocessing and to investigate the extraction and amplification of signals of interest by different band-pass filter bands, such as Movement-related cortical potentials (MRCP,0.05–6 Hz). In this study, We made preliminary band-pass pre-process for different frequency bands that may be triggered by MI, then the EEG signal was averaged over every 20 trials as “trial-block” to obtain a pronounced ERP curve. Pearson’s correlation coefficient was used for the functional connectivity estimation, directly expressing the correlation of amplitude characteristics. The Pearson correlation coefficient was calculated as follows:

(2) $$\begin{array}{c} \rho =\frac{E\left[\left(X-\mu_{X}\right)\left(Y-\mu_{Y}\right)\right]}{\sigma X\sigma Y}\\ \;=\frac{E\left[\left(X-\mu_{X}\right)\left(Y-\mu_{Y}\right)\right]}{\sqrt{\sum_{i=1}^{\text{n}}{\left(X_{i}-\mu_{X}\right)}^{2}}\sqrt{\sum_{i=1}^{\text{n}}{\left(Y_{i}-\mu_{Y}\right)}^{2}}} \end{array}$$

where *X* and *Y* represent the calculated signal values for trial-block ERPs of two channels. μ*X* and μ*Y* represent the mean of *X* and *Y*. σ*X* and σ*Y* represent the standard deviation of X and Y. The formula calculates the covariance ratio between the two channels to the product of two standard deviations.

#### Network Indicators

Graph theory plays a crucial role in network analysis. Each EEG channel represents a single node in graph. Degrees derived from Functional Connectivity estimates between nodes then form a graph. Since MI-action focuses on C3 and C4 nodes’ expression, we consider the direct calculation of the change in C3 and C4 nodes’ degree as the task proceeds.

(3) $$E_{i}\left(G\right)=\sum_{j\neq i\in G}d_{ij}$$

where *i* is the node of interest, *G* is the whole brain connectivity map. *J* is other nodes and *E_*i*_(G)* is the sum of the connection weights of the node of the claim. All other nodes within the region were calculated. We also performed the same calculation to O1 and P1 nodes’ degrees far away from the M1 region, used as a comparison study. Also, the summation of degrees for all nodes in the region provides a complete picture of the corresponding brain regions’ overall neural activity:

(4) $$E_{region}\left(R\right)=\sum_{j\neq i\in R}d_{ij}$$

Where *E*_*region*_ refers to the region of interest, which can be the left or right hemisphere. *R* is the set of nodes within the brain hemisphere, and *j* is the other nodes. This equation calculates the sum of the weights of all weighted edges in the region. This calculation allows us to estimate the overall activity of the nodes in the region.

The clustering coefficient (Gonzalez-Lima and Mclntosh, 1994; Latora and Marchiori, 2001) is used in this analysis, aiming to explore the whole brain’s variation. Clustering coefficients are divided into three calculation methods: global, local, and average. The global clustering coefficient is used to explore the variation of the whole brain. The clustering coefficient calculation requires that the graph be binary and coherent. Thresholds should be properly chosen to binarize the calculated connectivity in the brain network analysis. To ensure the connectivity of the graph, we use the threshold value of 0.6 in this experiment. The coefficient is obtained by dividing the number of closed-loop ternary groups by the number of all ternary groups in the graph, calculated as follows:

(5) $$C_{\text{total}}\left(G\right)=\frac{3\times G_{\Delta }}{3\times G_{\Delta }+G_{\Lambda }}$$

where *G*_Δ_ represents the number of closed-loop triads in the graph (three nodes connected), and *G*_*∧*_ means that there are only two edges with weight one between the three nodes. In this experiment, we calculate the changes of the clustering coefficients of the whole brain and the hemispheres overtime to get a global picture of the brain network’s changes in the short-term for the MI task.

## Results

### Change of ERD MuSC

**FIGURE 2:**
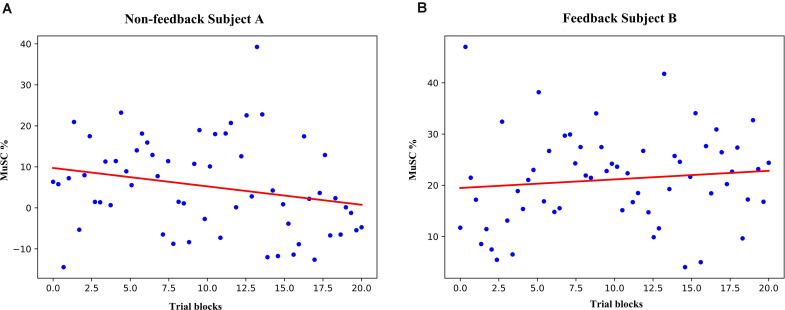
The MuSC of subject A and B. **(A)** the MuSC for non-feedback subject A (3-day experimental data are synchronized and averaged according to a set of 20 trials). The red line is a linear fit, where the slope of line A is negative (slope = −0.4). **(B)** the MuSC for Subject B, the slope of line B is positive (slope = 0.2).

We applied Mu-band boundary selection for each individual and results were within alpha and lower-beta band (5–20 Hz, filtering results listed in the Supplementary Material for reference). MuSC was calculated from the 636 non-feedback subject A and 616 feedback subject B trials then averaged for each consecutive 20 trials constructing “trial blocks” results. The MuSC of the non-feedback subject A tends to be downwards with the fitting line slope of −0.4 (Figure 2A), by contrast, the feedback B’s MuSC rises with the fitting line slope of 0.2 (Figure 2B), similar to the result in previous studies (Shindo et al., 2011; Yoshida et al., 2016). However, there is no significant difference between the two experiments due to the large variance. Changes in ERD’s MuSC can be observed in short-term MI experiments with feedback, but the trend of the data is subtle and difficult to use to measure the effect of short-term training. Four non-feedback subjects showed consistent result of ERD/ERS, thus we took non-feedback subject A as the typical subject to show the comparisons and analysis in the following.

### Change of ERP Network

We used different frequency bands (0.53–4 Hz, 3–6 Hz, 5–10 Hz, 8–16 Hz, and 15–30 Hz) to apply an initial inspection of degree-of-nodes for four non-feedback subjects (A,C,D,E) and 3-day data separately of the feedback subject B. This was to design an appropriate EEG preprocessing filter before construction the ERP functional network. We study the three sequential sessions with MI training of both paradigms and label the first to third sessions in the experimental sequence as super-trial 1–3, respectively. Results showed that both the 2nd and 3rd super-trial of the 3-day data of the feedback group in 3–6 Hz had significant decrease compared to the 1st super-trial (*p* = 2e-3, 7e-3 for day 1 respectively, *p* = 2e-3, for day 2, *p* = 6.5e-4, 5e-5 for day 3, respectivley) (see Table 1). Some other frequency band above 5 Hz also showed a partially significant trend. However, signal in the 0.53∼4 Hz band of the low frequency component did not show differences during on-going training sessions. In contrast, non-feedback subjects showed subtle increase at 0.53∼4 Hz, while no trend showing in other frequency ranges. Results indicated a consistent change along the short-term training for the feedback group.

**TABLE 1:** Degree-of-nodes for all subjects in different frequency bands.

Freq	0.53∼4 Hz			3∼6 Hz			5∼10 Hz			8∼16 Hz			15∼30 Hz		
Subj	Sess1	2	3	Sess1	2	3	Sess1	2	3	Sess1	2	3	Sess1	2	3
*B1*	29.3 ± 0.8	28.3 ± 2.6	28.5 ± 1.9	**30.4 ± 0.6**	28.1 ± 1.4**	27.1 ± 3.0**	23.6 ± 2.8	24.3 ± 1.9	21.9 ± 4.1	19.5 ± 2.7	22.1 ± 1.9*	20.3 ± 3.1	20.2 ± 2.2	18.6 ± 2.1	19.6 ± 2.2
*B2*	**29.7 ± 0.9**	**30.3 ± 0.6**	29.2 ± 1.0**	**29.0 ± 0.9**	27.6 ± 1.4*	**25.7 ± 2.6**	21.4 ± 3.4	21.7 ± 3.4	20.2 ± 3.0	19.5 ± 4.2	19.2 ± 2.3	20.6 ± 2.4	16.3 ± 2.7	16.8 ± 2.8	17.4 ± 1.6
*B3*	30.6 ± 0.6	29.6 ± 1.9	29.7 ± 1.7	**30.5 ± 0.9**	27.1 ± 1.4**	25.8 ± 1.5**	**25.0 ± 2.3**	**23.2 ± 2.2**	22.2 ± 2.2*	23.7 ± 2.2	21.9 ± 2.6	21.7 ± 2.7	**18.5 ± 1.6**	**20.9 ± 1.1^++^**	**19.0 ± 2.6**
*A*	18.8 ± 3.1	16.3 ± 2.5	17.4 ± 3.6	20.0 ± 1.9	17.5 ± 2.8	18.8 ± 2.3	17.7 ± 1.2	18.5 ± 1.7	18.7 ± 2.5	15.0 ± 1.7	16.0 ± 1.9	16.6 ± 1.5	14.8 ± 1.7	14.8 ± 1.1	15.3 ± 1.1
*C*	**27.1 ± 1.2**	**27.0 ± 1.3**	**28.2 ± 1.2^+^**	25.9 ± 1.7	25.2 ± 2.1	25.4 ± 1.1	26.1 ± 1.0	25.7 ± 1.4	26.1 ± 1.0	21.9 ± 1.4	21.4 ± 1.9	20.8 ± 0.8	16.7 ± 1.3	16.0 ± 1.6	15.7 ± 1.4
*D*	**26.9 ± 1.7**	**28.5 ± 1.1**	**28.1 ± 0.9^+^**	24.4 ± 1.6	23.3 ± 1.3	22.7 ± 2.0	21.6 ± 1.5	20.0 ± 1.8	20.6 ± 1.1	17.7 ± 1.8	16.7 ± 2.3	17.1 ± 1.5	14.3 ± 0.9	14.4 ± 1.3	14.9 ± 2.1
*E*	**20.7 ± 2.6**	**21.6 ± 4.1**	**22.4 ± 1.5^+^**	22.1 ± 2.3	23.7 ± 2.1	22.5 ± 1.1	16.0 ± 1.8	14.5 ± 1.5	15.7 ± 2.1	17.7 ± 1.2	16.7 ± 1.6	17.5 ± 1.2	16.3 ± 1.1	17.1 ± 1.3	16.1 ± 1.4

*Decreasing with p < 0.05; **: decreasing with p < 0.01.,+: increasing with p < 0.05; ++: increasing with p < 0.01. The bold values means the significant change of the data (p < 0.05 or p < 0.01).

**FIGURE 3:**
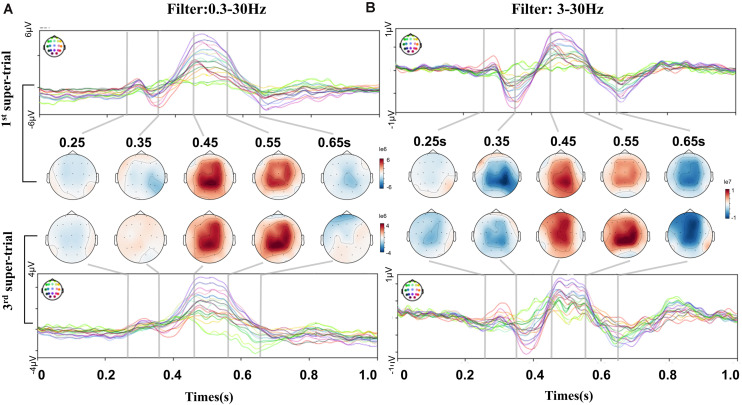
ERP and topographic comparisons between the 1st and 3rd super-trials of the short-term BCI training process. This comparison was for feedback subject B. Each super-trials containing consecutive 100 non-hold trials. **(A)** Filter with 0.3–30 Hz. No significant change between the 1st and 3rd super-trials. Some drift changes were present in the prefrontal channels. **(B)** Filter with 3–30 Hz. The 1st and 3rd topographic maps show dynamic differences. N-potential attenuation at 0.35 s, P-potential enhanced at 0.55 s, then N-potential enhanced at 0.65 s.

To investigate the detailed dynamic change along the short-term training, we compared 1st and 3rd super-trial ERPs of the feedback subject B, at both 0.3–30 Hz and 3–30 Hz frequency bands. In the case of 0.3–30 Hz filtering (Figure 3A), the ERP dynamic processes did not show significant changes, with topographic maps appearing similarly patterns at the MI task. However, ERP dynamic changes were revealed under the 3–30 Hz filtering (Figure 3B), such as a strengthening of the negative potential at 0.35 s, of the following positive potential at 0.55 s, and the negative potential at 0.65 s. ERP features at 3∼30 Hz presented strengthened deflections from the beginning of the training session to the end. By combining results in Table 1, result indicated the EEG low frequency component containing MI brain activities, as consistent with (Ramos-Murguialday and Birbaumer, 2015; Schwarz et al., 2019), and it contained information of short-term variations at the feedback paradigm. We choose a 3∼30 Hz band-pass filter to capture EEG characteristics as interested before average and further analysis.

**FIGURE 4:**
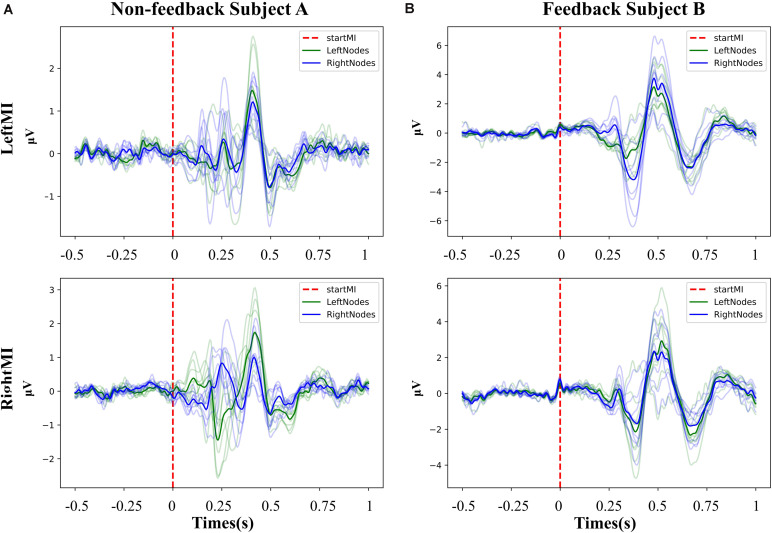
The overall ERP performance of the feedback and non-feedback subject. **(A)** Non-feedback subject A, the potential graph of each channel during left- and right- handed MI training (−0.5∼1 s). **(B)** Feedback subject B, the potential graph of each channel during left- and right- handed MI training (−0.5∼1 s). Both subjects present clear ERP curves, and the ERP curves of the left channels and the right channels show slight differences at different MI task.

As event-related responses apart from Mu-suppression, we analyzed ERPs of left or right MI task for non-feedback subject A and feedback subject B. It was derived from averaging 20 trials at −0.5∼1 s filtered at 3–30 Hz for of all EEG channels. The ERP responses initiated after the start of the MI task. The overall ERP performance of the feedback (Figure 4A) and non-feedback subject (Figure 4B) were stable for left- and right-handed MI, with slightly different ERP performance for different side-channels for left- and right-handed MI.

#### Degree of Nodes

**FIGURE 5:**
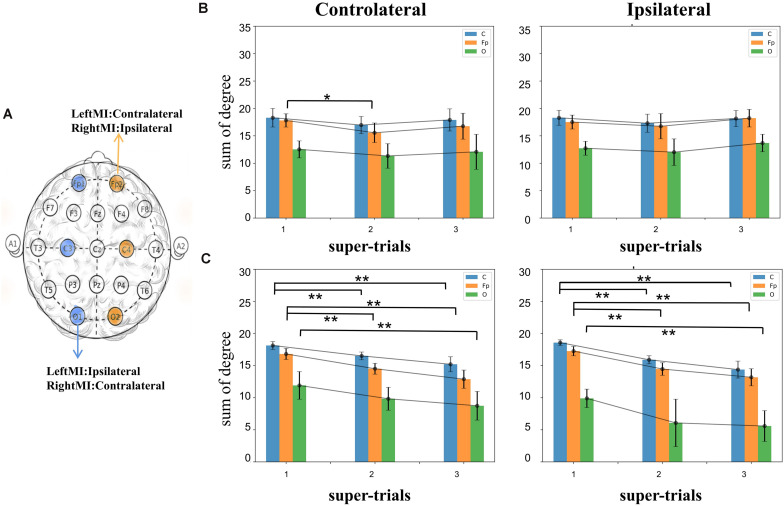
**(A)** A schematic representation of the one-node degree analysis. **(B)** Single node degree after averaging the three non-feedback trials of subject A, the effect tends to be smooth, where the contralateral Fp node degree shows a significant change of 1–2 super-trial (*t*-test value *p* < 0.05). **(C)** The single node degree of subject B, both the ipsilateral and contralateral single nodes have a decrease relative to the initial value (*t*-test value C’s ipsilateral:*s* = 5.60, *p* < 0.01; *s* = 2.97, *p* < 0.01, C’s contralateral: *s* = 10.40, *p* < 0.01, *s* = 3.13, *p* < 0.01, Fp’s ipsilateral: *s* = 5.69, *p* < 0.01, *s* = −7.09, *p* < 0.01, Fp’s contralateral: *s* = 6.85, *p* < 0.01, *s* = −8.08, *p* < 0.01). The symbols * and ** represent the mark of significant and very significant changed data.

Single node degrees were analyzed for non-feedback subject A and feedback subject B, respectively. Figure 5A shows the analyzed nodes. The contralateral analysis target nodes for left-handed motion include C4, O2, Fp2, and right-handed C3, O1, Fp1, and the opposite nodes for ipsilateral motion. The subject experiments were divided into three groups according to the order in which the sessions were performed. The trials for MI task execution were selected from each group, averaging the 20 original trials to containing ERP features to calculate the network’s connectivity. In Figure 5B, the connectivity histogram of subject A’s ipsilateral and contralateral sides Fp node’s contralateral side is significantly different (*t*-test value *p* = 0.01) between the first and third super-trials, while the other nodes not significantly different. In Figure 5C for subject B, the second and third super-trials of the C and Fp nodes are significantly different from the first in both ipsilateral and contralateral (*t*-test value *p* = 2.3e-5, 1.2e-5 for C; *p* = 2.1e-5,3.7e-6for Fp in contralateral and *p* = 1.5e-8, 2.5e-6 for C; *p* = 3.2e-6,1.2e-6 for Fp in ipsilateral); in addition, the O nodes’ contralateral experiments were significantly different between the first and third super-trials (*t*-test value *p* = 0.6e-2 and 0.3e-3 in contralateral and ipsilateral). Different significant downward trends can be observed in subject A and subject B. Thus, we hypothesize that feedback BCI training leads to decreased node degrees in the ERP brain network.

#### Degree of Region

**FIGURE 6:**
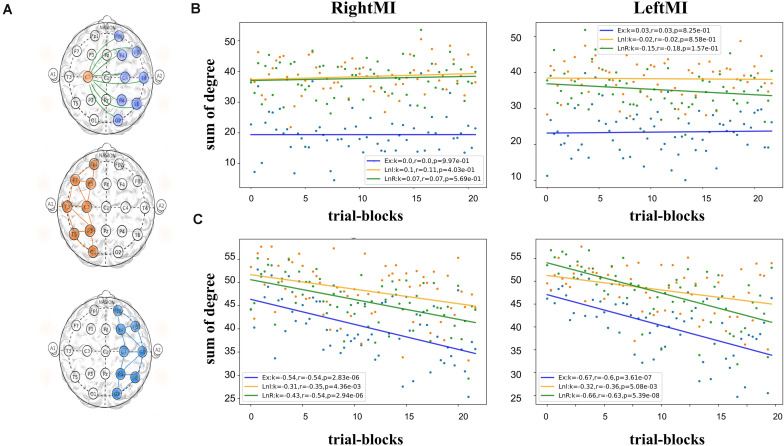
**(A)** A schematic representation of the nodes included in the three computational methods, from top to bottom, Ex, LnL, and LnR. **(B)** Scatter plot of the brain network indicators in the MI task state of Subject A and calculates the linear regression fitted straight lines for the three scatter types. Among them, B-figure left EX,LNL,LNR; **(C)** Scatter plots of network indicators in subject B’s feedback experimental data, and the slopes of all straight lines fitted are negative, **(B,C)** indicate the gradients of LnR in their leftMI are all less than LnL.

In this part, we calculated the sum of the connectivity in the left and right hemispheres as LnL and LnR, and connectivity between two sides (excluding the medial node) as EX (Figure 6A). Then used linear regression to fit a straight line of scatter. In Figure 6B for subject A, The slopes of the three fitted lines all approach 0 in both left-handed and right-handed MI. In Subject B’s feedback experiment (Figure 6C), the slopes of all fitted lines were negative, indicating a decrease in regional connectivity. During left-handed MI, the slope of LnR on the opposite side was smaller than that of LnL on the same side (Ex fitting line slope = −0.67, Lnl fitting line slope = −0.32, LnR fitting line slope = −0.66), whereas this phenomenon does not appear, the fitted lines for right-handed MI are (Ex fitting line slope = −0.53, Lnl fitting line slope = −0.31, LnR fitting line slope = −0.42). Figure 5 gives a clear contrast between the regional degree summation. The feedback experiments will have an overall downward trend, and its contralateral downward trend is more pronounced in left MI. The slopes of LnR in their leftMI are smaller than LnL both in subjects A and B, which is presumed to be related to the ERD/ERS features of the EEG.

#### Clustering of Network

**FIGURE 7:**
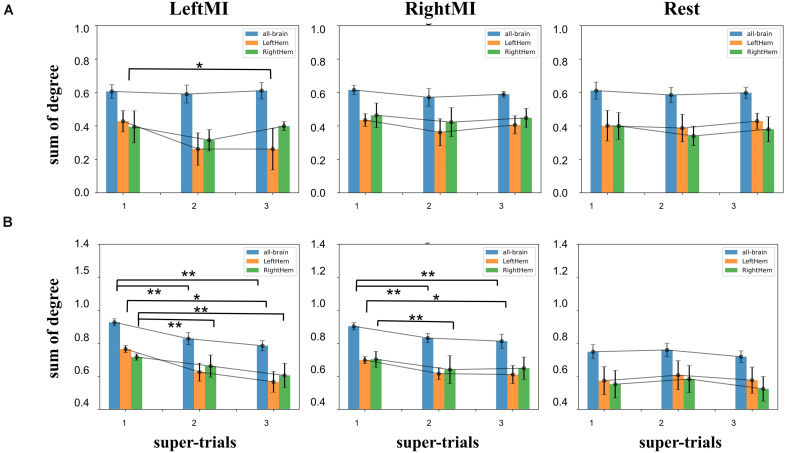
**(A)** Clustering coefficients histograms of non-feedback subject A, left, middle and right plots were calculated for left-handed MI, right-handed MI, and rest condition, a significant decrease in the right hemispheric region value in 1–2 trials during left-handed MI (*t*-test value *p* < 0.05), rest condition The all-brain indicator was also significantly different (*t*-test value *p* < 0.05); **(B)** clustering coefficients of feedback subject B, there was an extremely significant downward trend in the left-handed MI for both the all-brain and right hemisphere indicators 1–2, 1–3 (*t*-test value *p* < 0.01), left hemisphere had an extremely significant difference only between 1 and 3 experimental comparisons (*t*-test value *p* < 0.05). In rightMI, all-brain had a significant decrease between 1 and 2, 1 and 3 super-trials (*p* < 0.01). Left hemisphere and right hemisphere indicators have significant changed between 1 and 3 super-trials (*p* < 0.05) and 1–2 super-trials (*p* < 0.01), respectively. The symbols * and ** represent the mark of significant and very significant changed data.

Clustering coefficients were calculated for the whole brain, left hemisphere, and right hemisphere. The differences between the task and resting states were calculated separately. Among the three calculations of subject A (Figure 7A), there was a downward trend and significant difference (*t*-test value *p* = 0.04) between the 1–3 super-trials of left-handed MI in the right hemisphere. Figure 7B for Subject B shows a significant downward trend for left-handed MI’s both all-brain and right hemisphere (*p* = 1.3e-3 and 0.2e-3 for all-brain), and the left hemisphere was significantly different only in first-to-third experimental comparisons (*t*-test value *p* = 0.04); in right-handed MI, all-brain, left and right hemisphere had significant decreases (all:*t*-test value *p* = 4.3e-3, 6.9e-3, left: *t*-test value *p* = 0.04,right:*p* = 4.3e-3) while there are no significant changes in the rest state. The results show that feedback experiments altered the task-state clustering coefficient to decay and more extensive in the contralateral sides. Training did not affect the resting state significantly.

## Discussion

In the present study, we focused on using EEG signals to investigate what impact MI-BCI training can have on the brain in short-term. We applied controlled research using MI-BCI training with/without visual feedback.

Firstly we analyzed the Mu band’s energy attenuation on the contralateral side. The result showed ERD changed with an increasing trend at the feedback group. This was consistent with studies of rehabilitation in Shindo et al. (2011) and Yoshida et al. (2016), suggesting ERD strengthened for successful BCI training. On the other hand, the non-feedback group presented little change, and the change from 1-h feedback training was of no statistical significance, which was different from the long-term rehabilitation training. Therefore, characteristics of cortical motor activities need to be further investigated, to introduce new assessment tools to quantify changes with MI-BCI training of short-terms.

We then studied ERPs of MI tasks in this study. In the MI analysis of ERP, MRCP is often used. the ERP analyzed in this paper intersects with MRCP but is not identical in definition. The low frequency (below 6 Hz or so) negative shifts in the EEG signal representing brain activity changes related to movement. In our investigation, the negative deflection of MRCP appeared relatively obvious only after filtering above 3 Hz. The corresponding ERP dynamic presented visible changes along the MI training process as well. The EEG signal band-pass filtered at 3–6 Hz contains information of significant changes in relation to short-term training. On the other hand, signals below 3 Hz had relatively large amplitude but the response was dynamically consistent during the training process. This may obscure functional changes of great interest to us. Previous studies have mentioned that there are discriminable information for MI decoding in Bands at 1∼6 Hz of ERP (Ramos-Murguialday and Birbaumer, 2015; Korik et al., 2018; Schwarz et al., 2019). For example, Ramos et al. used filtering in the 3∼45 Hz for a BCI motor task analysis. Korik et al. studied ERP at 4∼8 Hz for decoding imagined 3D hand movement in EEG (Korik et al., 2018). Marshall et al. investigated ERP with 3 Hz high-pass filter for infants’ auditory (Marshall et al., 2009). Thus we applied preprocess filtering with low cut-off frequency at 3 Hz to satisfy our analysis requirements. As we choose 3–30 Hz of EEG containing MI brain activities for investigation functional changes during short-term training, it contains ERD range as well.

Different behavioral patterns have different brain network activations (Gonzalez-Lima and Mclntosh, 1994; Büchel and Friston, 1997; Büchel and Friston, 2000; Horwitz et al., 2000; Taylor et al., 2000). Functional connectivity has been defined as ‘neural context’ (McIntosh, 1999, 2000; McIntosh et al., 2001). By calculating functional connectivity, we can further apply graph theory to analyze brain networks. Graph theory, which describes the brain as a single interconnected network (Bullmore and Sporns, 2009; Fallani et al., 2013; Cheng et al., 2015), provides a theoretical framework with the potential ability to characterize the behavior of complex brain systems and can reveal important information about the local and global organization of functional brain networks. Applying the methodology described above, this paper validates the changes in brain networks brought about by short-term MI training of these two neural contexts with and without feedback and their differences. For example, in Figure 5, we see that the feedback experimental set of individual nodes of this brain network (i.e., with visual feedback) shows a significant downward trend in degree summation. We speculate that this trend stems from the fact that MI training with visual feedback leads to decreased connectivity of the blocks represented by the nodes due to stronger inhibitory action generation, mentioned in previous literature (Waldvogel et al., 2000; Attwell and Iadecola, 2002). In Figure 7, we analyzed network connectivity changes from the perspective of the cerebral hemispheres’ internal and external interactions. We speculated that the decreasing trend of the feedback group might be caused by the concentration of neural clusters in the brain area and the concentration of ERP changes in specific relevant areas, which led to a decrease in the overall correlation within the region. The reduction in the contralateral MI of the left hand indicated certain ERD characteristics. Feedback MI training more significantly affects brain networks in the task state than in the resting state. In conclusion, this ERP-based constructed network change showed a significant decrease in the short-time task state, contralateral effectiveness, etc., intuitively reflecting the immediate effect of short-term BCI training on the brain.

In the current research on BCI rehabilitation training, we see that many studies have been devoted to finding indicators of long-term rehabilitation. In contrast, the indicators proposed in this paper found that brain network activity changes over a short period. Feedback training results are more significant than those of no-feedback training indicators, which are expected to be applied to short-term training value assessment. Unlike other classical brain network analysis methods such as fMRI (Van Den Heuvel and Pol, 2010), EEG signals have unique advantages—high temporal and spatial resolution, which can be analyzed more quickly and easily. It makes a good pavement for the short-term MI-BCI rehabilitation assessment. This paper differs from the conventional brain network construction of EEG (Friston, 2011). It adopts an EEG signal combination processing method with ERP characteristics, which can reduce EEG signals’ instability and reflect signals’ event characteristics more effectively.

However, there are many limitations for improvement in this study. For example, the experimental sample data is insufficient. The ERP construction method used for network construction has not been tried in non-MI rehabilitation training. The present analysis is based on the visual feedback training of healthy subjects. The sample data can be improved in many aspects: for example, changing healthy subjects to patients or using different feedback methods; it is also possible to make a comparison between short-term training and long-term training indicators and integrate the processes of existing indicators proposed in this paper to form a perfect evaluation method to quantify the goals of rehabilitation training better.

## Conclusion

In summary, this paper is a preliminary attempt in the field of EEG brain network-based rehabilitation assessment. We applied Mu band power’s attenuation and ERP-based brain network to analyze the EEG changes during short-term MI task. We found significant changes in brain connectivity, that the functional network topology coefficients of feedback subject showed a significant decrease after about 1 h of MI-BCI training, while the non-feedback group’s most network parameters didn’t change significantly. The experimental results showed the necessity of neurofeedback. This study has laid a good foundation for subsequent BCI closed-loop neurological rehabilitation studies. The analytical approach for measuring the effectiveness of short-term rehabilitation training proposed in this study is expected to facilitate the establishment of a more personalized rehabilitation assessment system, which, when correlated with long-term clinical indicators, can lead to more credible and regulated individual treatment schedules and help patients to undergo more efficient rehabilitation.

The next step of the study is to collect more data or try to apply generative methods to deal with the data scarcity. Furthermore, we also consider different feedback strategies to link the short-term indicators to the specific neurological mechanisms, so as to provide a more underlying and reliable basis for experimental results.

## Data Availability Statement

The original contributions presented in the study are included in the article/Supplementary Material, further inquiries can be directed to the corresponding author/s.

## Author Contributions

YW: conceptualization. YW and JL: validation and writing—original draft preparation. JL, YW, and QC: formal analysis. QC and HW: resources. JL, QC, and HW: writing—review and editing. All authors have read and agreed to the published version of the manuscript.

## Conflict of Interest

The authors declare that the research was conducted in the absence of any commercial or financial relationships that could be construed as a potential conflict of interest.
