# Supplementary figures and images for: Changes in EEG Brain Connectivity Caused by Short-Term BCI Neurofeedback-Rehabilitation Training: A Case Study
Source: Front Hum Neurosci. 2021 Jun 24;15:627100. doi: 10.3389/fnhum.2021.627100 (PMC8336868; doi:10.3389/fnhum.2021.627100)

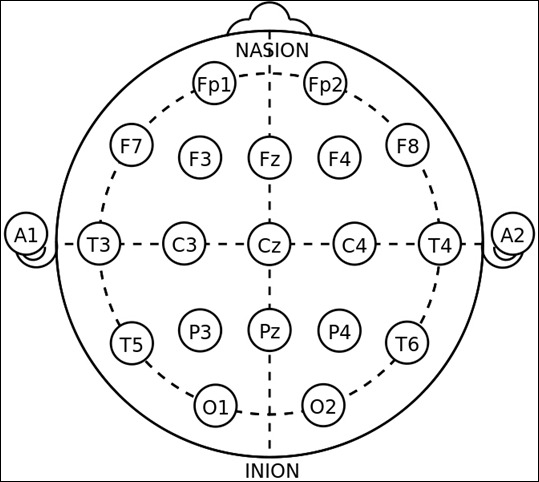

Supplement: Supplementary file 1 [file Image_1.JPEG]
