# Supplementary material for: Changes in EEG Brain Connectivity Caused by Short-Term BCI Neurofeedback-Rehabilitation Training: A Case Study
Source: Front Hum Neurosci. 2021 Jun 24;15:627100. doi: 10.3389/fnhum.2021.627100 (PMC8336868; doi:10.3389/fnhum.2021.627100)
Supplement: Supplementary file 2 [file Presentation_1.PDF]

1. **Mu-band boundary of all subjects in three days:**

Table S1.Mu-band boundary of all subjects in three days

**Subj/day**	**A/1**	**A/2**	**A/3**	**B/1**	**B/2**	**B/3**	**C/1**	**C/2**	**C/3**	**D/1**	**D/2**	**D/3**	**E/1**	**E/2**	**E/3**
**Freq(Hz)**	14-17	12-15	12-15	17-20	17-20	16-19	12-15	17-20	17-20	17-20	17-20	17-20	17-20	12-15	12-15
**Time(s)**	0.5-1	0.5-1	0.5-1	0.1-0.6	0.1-0.6	0-0.5	0.5-1	0.4-0.9	0.5-1	0.5-1	0.5-1	0.5-1	0-0.5	0-0.5	0.5-1

By comparing the band energy with that of the resting state, we screened for the frequency time period in which each subject showed the most significant ERD performance in a single day. These results will be used for subsequent analyses.

2. Degree of nodes results in different filter ranges

Use degree of nodes to filter suitable ERP observation ranges. The figure shows that there is a significant decrease in the results of subject B with feedback in the case of 3-6 Hz filtering over 3 days.

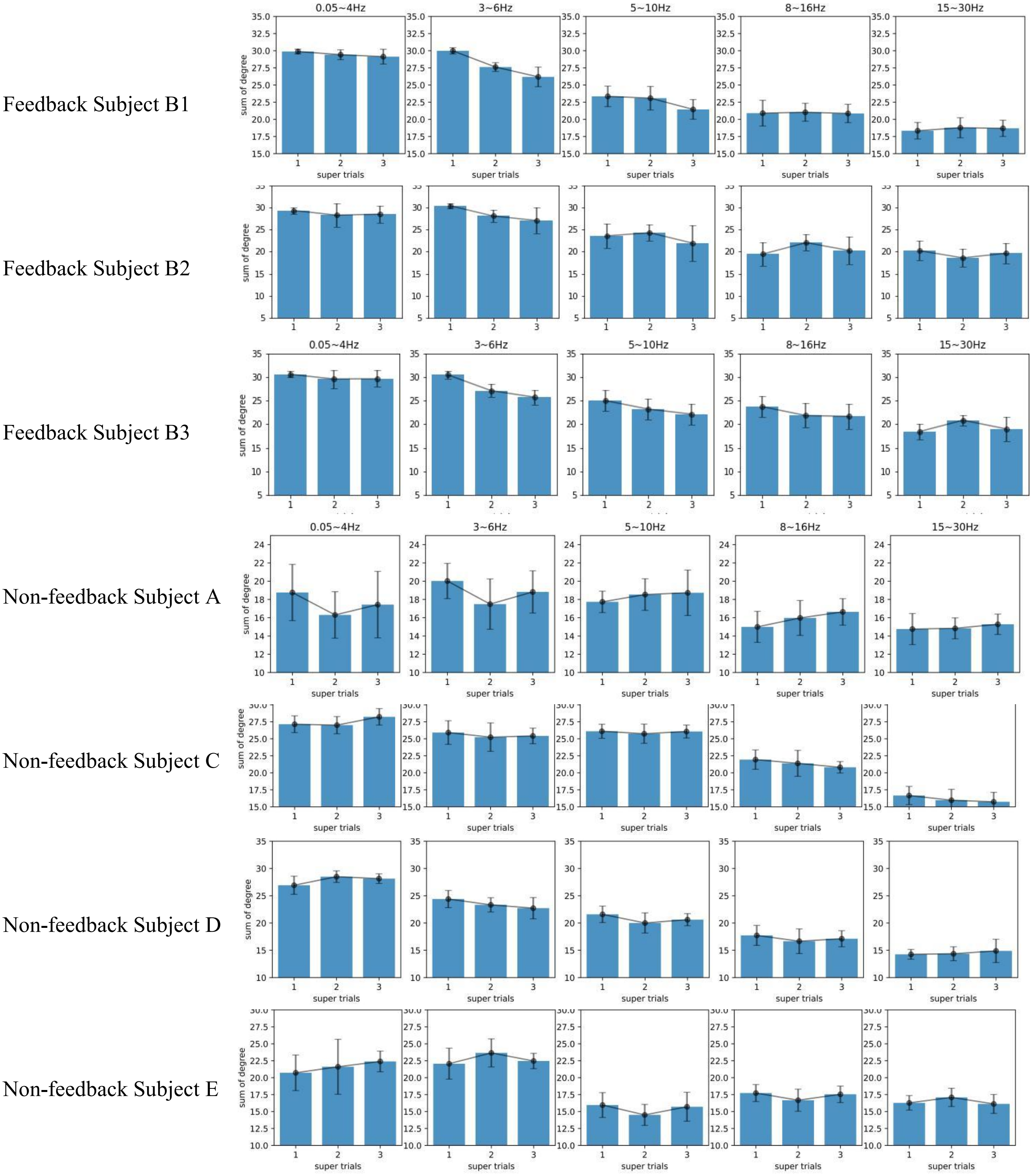

Figure S1. Degree of nodes results in different filter ranges

3. Network results of other non-feedback subject

We present the results of network analysis for subjects other than A. Similar to the results of A, there is no significant trend for other non-feedback subjects

Non-feedback Subject C

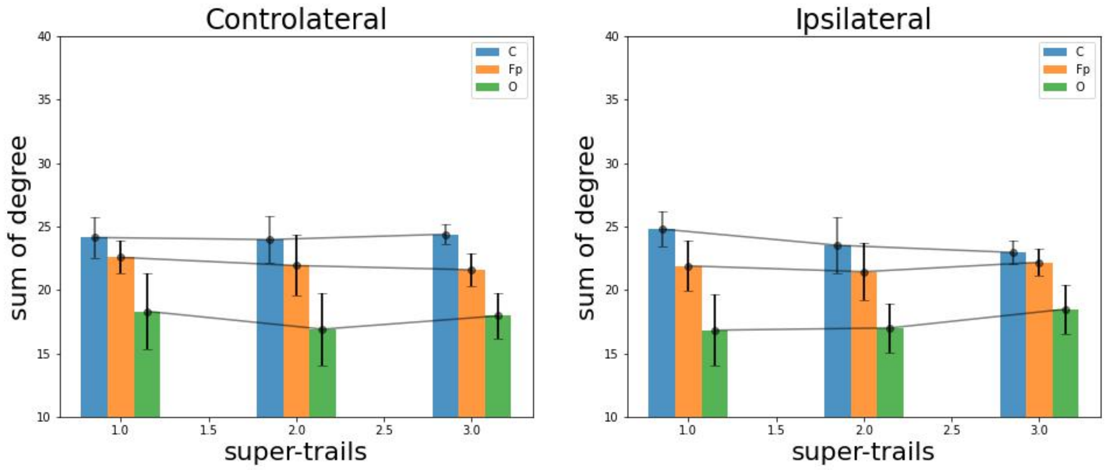

Degree Of Nodes

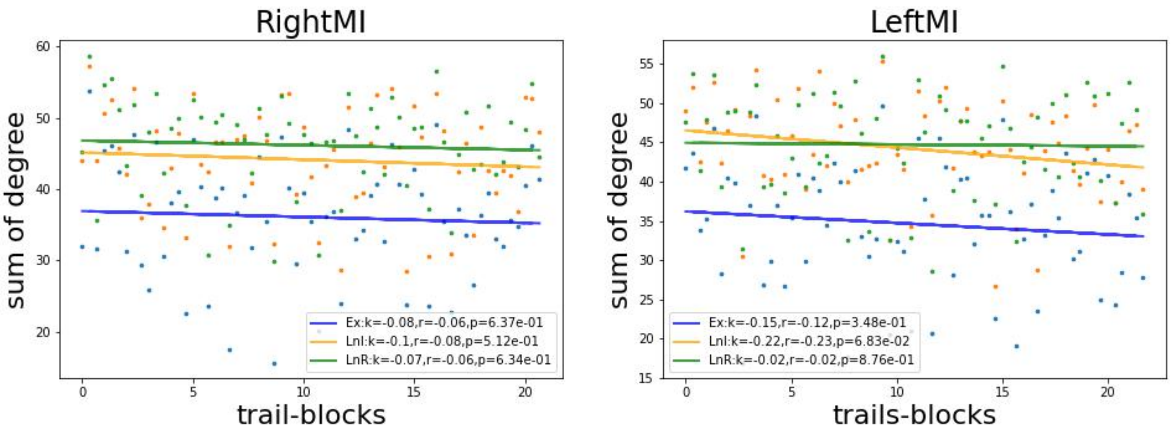

Degree Of Regions

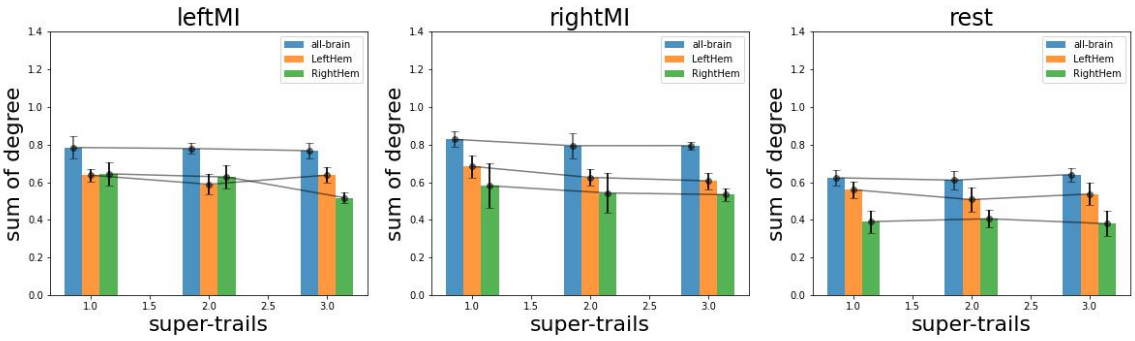

Clustering coefficients

Figure S2. ERP network analysis for non-feedback Subject C

Non-feedback Subject D

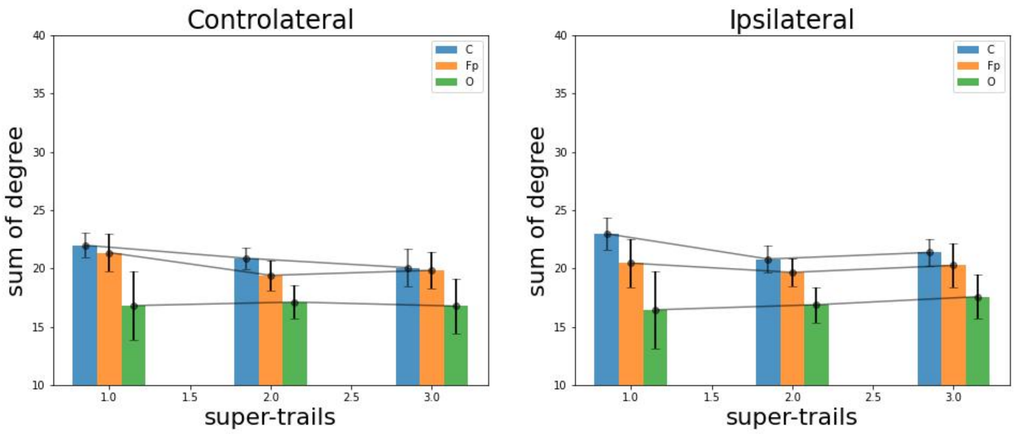

Degree Of Nodes

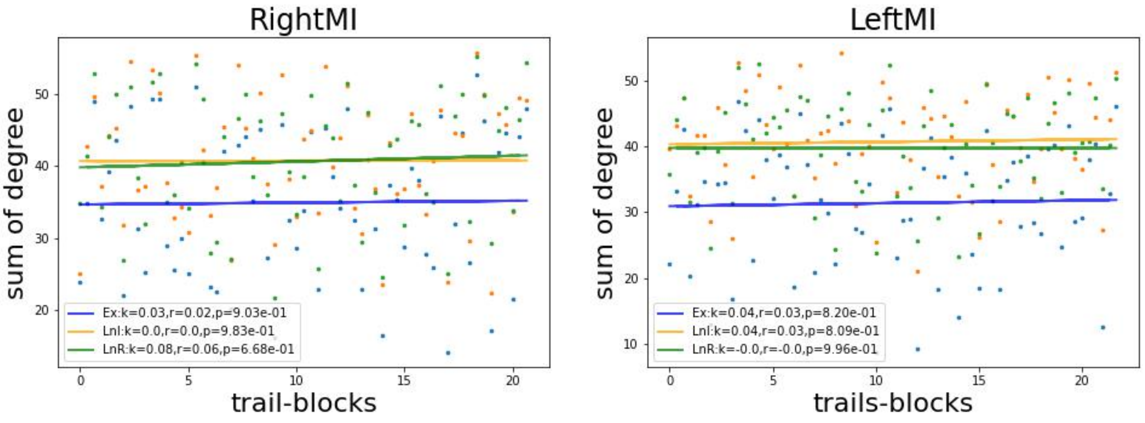

Degree Of Regions

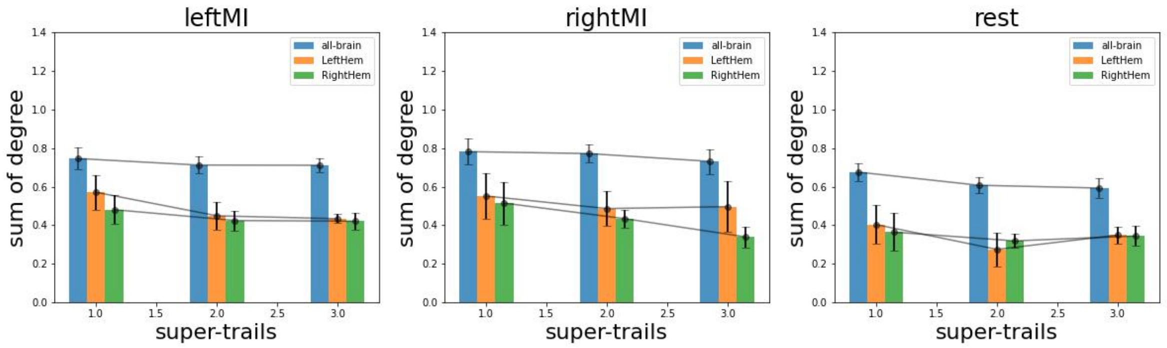

Clustering coefficients

Figure S3. ERP network analysis for non-feedback Subject D

Non-feedback Subject E

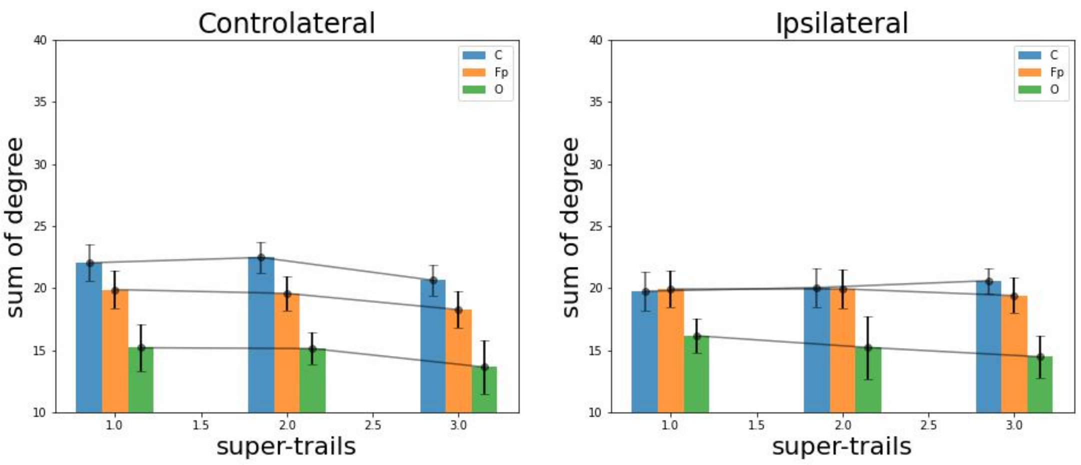

Degree Of Nodes

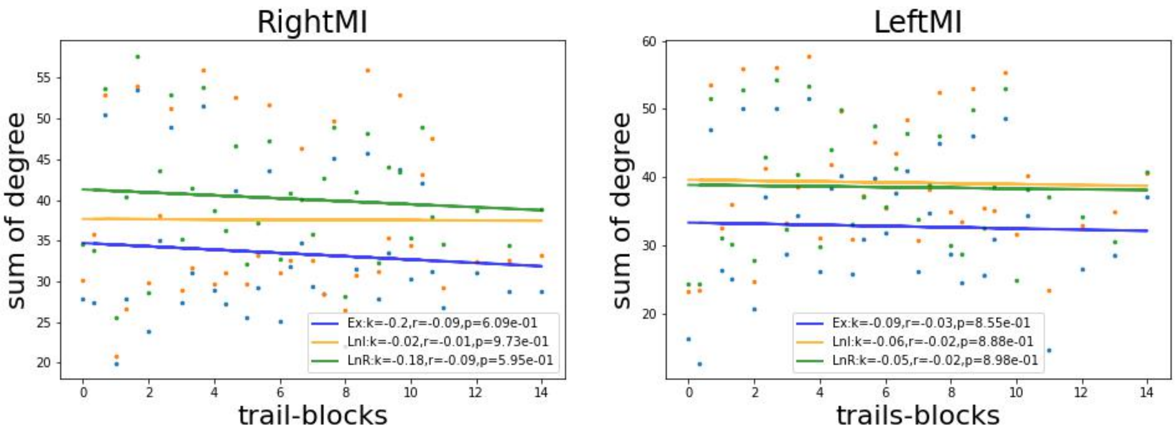

Degree Of Regions

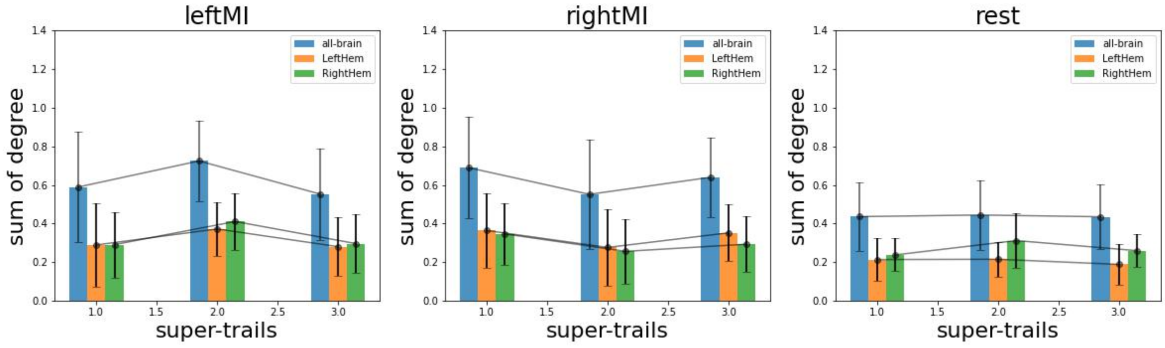

Clustering coefficients

Figure S4. ERP network analysis for non-feedback Subject E

4. ERP filter range comparison

We compared the ERP curves for all subjects with 3~6 Hz filtering and 3~50 Hz filtering. figure shows that the 3~50 Hz filtering presents more detail without causing major disturbances to the overall ERP curve.

Non-feedback subject A:

3~6Hz

3~50Hz

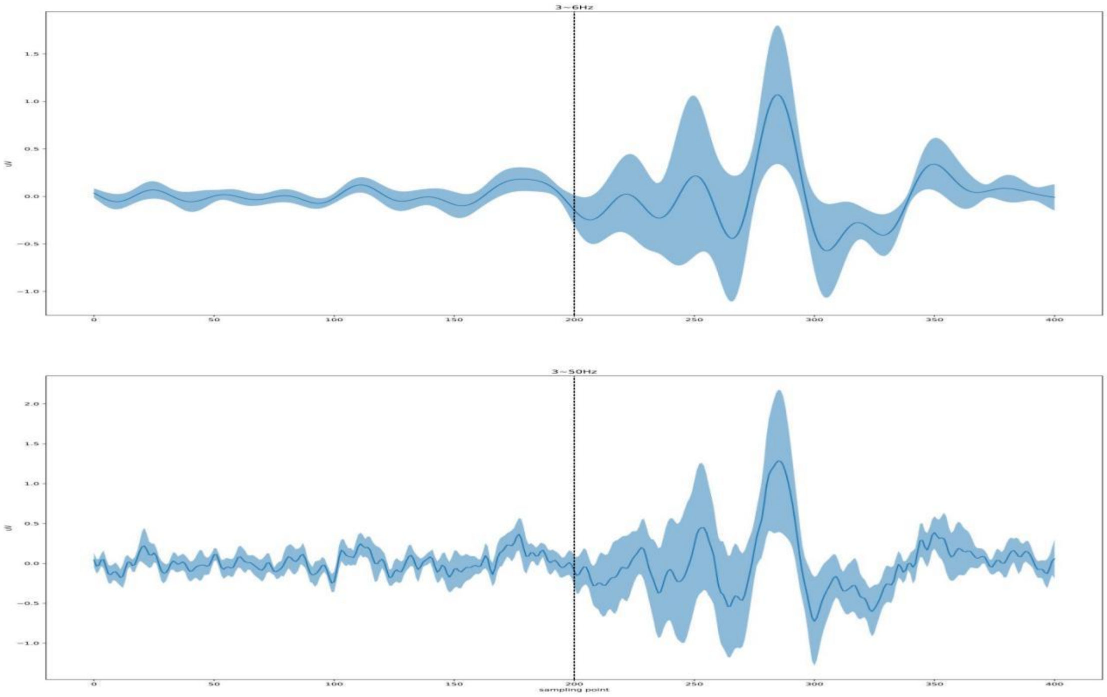

Figure S5. ERP curves analysis for non-feedback Subject A

Feedback subject B:

3~6Hz

3~50Hz

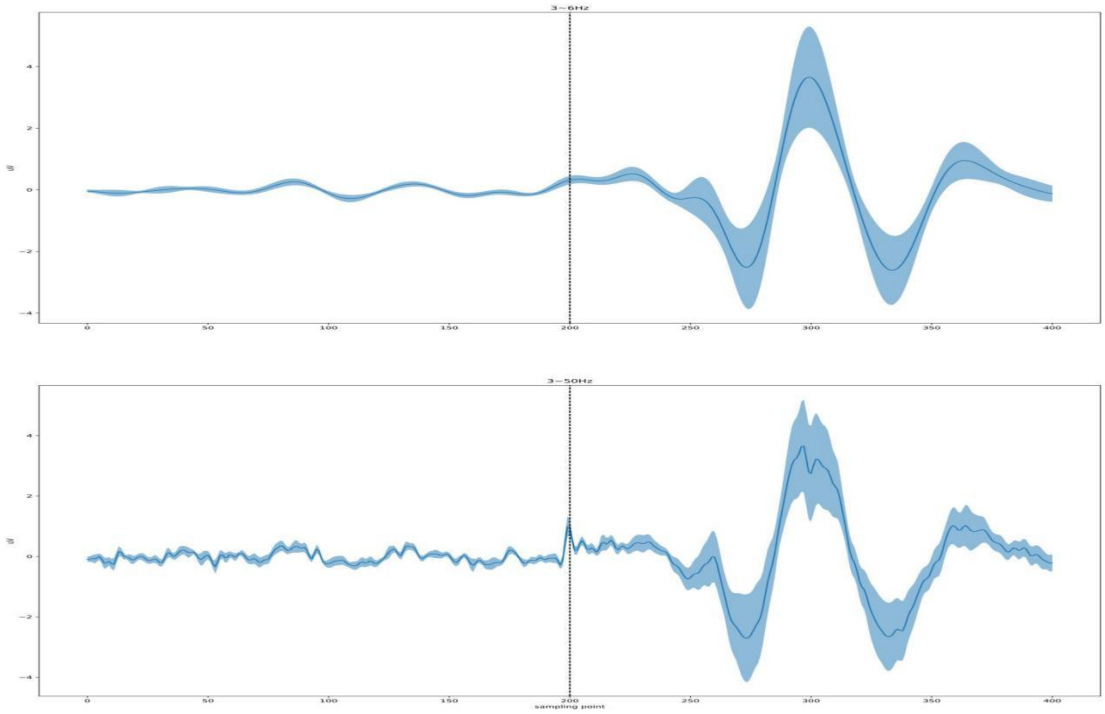

Figure S6. ERP curves analysis for feedback Subject B

Non-Feedback subject C:

3~6Hz

3~50Hz

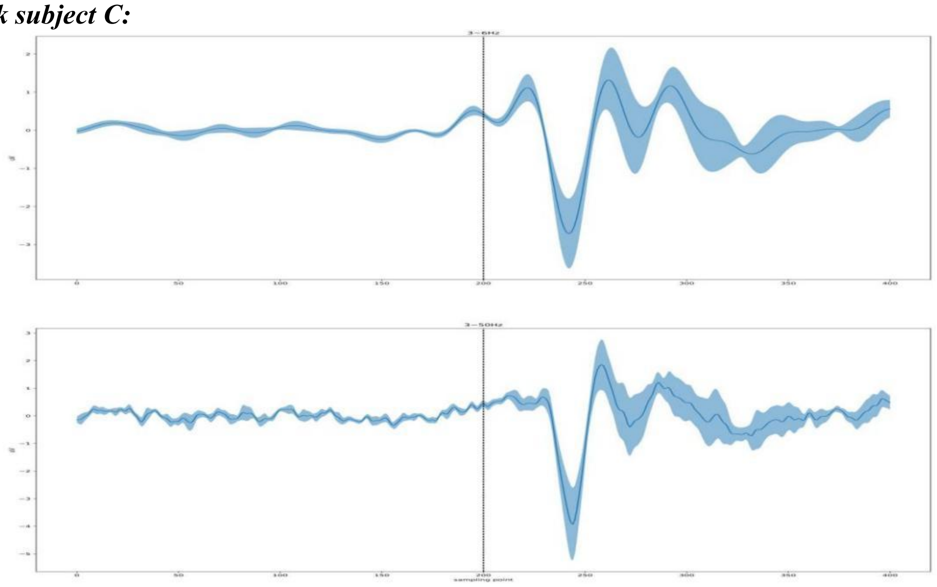

Figure S7. ERP curves analysis for non-feedback Subject C

Non-Feedback subject D

3~6Hz

3~50Hz

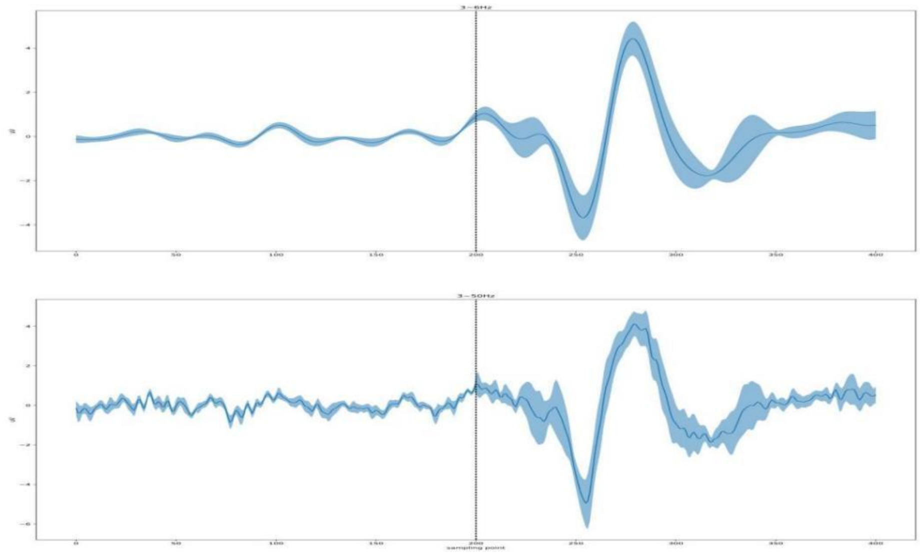

Figure S8. ERP curves analysis for non-feedback Subject D

Non-Feedback subject E

3~6Hz

3~50Hz

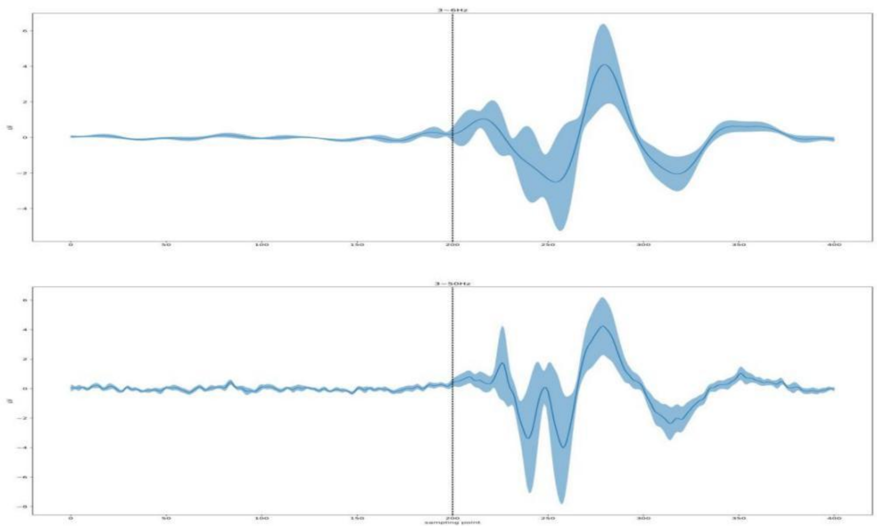

Figure S9. ERP curves analysis for non-feedback Subject E
