# Supplementary material for: Changes in EEG Brain Connectivity Caused by Short-Term BCI Neurofeedback-Rehabilitation Training: A Case Study
Source: Front Hum Neurosci. 2021 Jun 24;15:627100. doi: 10.3389/fnhum.2021.627100 (PMC8336868; doi:10.3389/fnhum.2021.627100)
Supplement: Supplementary file 3 [file Presentation_2.PDF]

The original dataset only shared the gender and age of the subjects, and no other information was given as they were healthy subjects.

Here follows subject's information:

SUBJECTA - Male, 20-25 y.o.

SUBJECTB - Male, 20-25 y.o. non-feedback

SUBJECTC - Male, 25-30 y.o. non-feedback

SUBJECTD - Male, 25-30 y.o.

SUBJECTE - Female, 20-25 y.o. non-feedback

SUBJECTF - Male, 30-35 y.o. non-feedback

SUBJECTG - Male, 30-35 y.o.

SUBJECTH - Male, 20-25 y.o.

SUBJECTI - Female, 20-25 y.o.

SUBJECTJ - Female, 20-25 y.o. feedback

SUBJECTK - Male, 20-25 y.o.

SUBJECTL - Female, 20-25 y.o.

SUBJECTM - Female, 20-25 y.o.

Subjects marked in blue have complete data from 3 experiments, so they were used in our study.

Subject B,C,E,F are non-feedback subjects B,C,D,E in our manuscript(where the results for C,D,E are similar to those for B and have been included in the additional material).

Subject A is the only publicly available experimental subject with feedback in the original dataset.
