# Supplementary material for: Changes in EEG Brain Connectivity Caused by Short-Term BCI Neurofeedback-Rehabilitation Training: A Case Study
Source: Front Hum Neurosci. 2021 Jun 24;15:627100. doi: 10.3389/fnhum.2021.627100 (PMC8336868; doi:10.3389/fnhum.2021.627100)

ERD Mu band selection

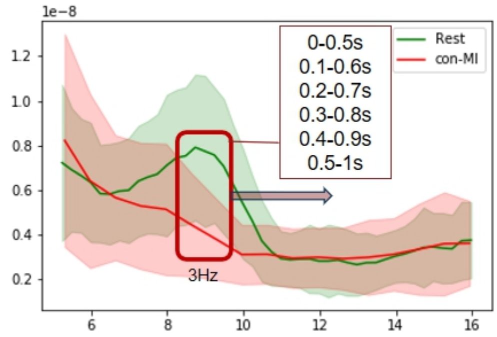

Each trial was screened for ERD using a sliding window, and the most pronounced segments were selected for statistics.

ERP selection

Here follows the average of 900 trials for subject A. We can see a clear ERP curve (filtered from 3-50 Hz).The subsequent analysis intends to use such generalized ERP features for brain network construction, so a certain number of trials need to be averaged so that a small number of trials can be combined to produce a clear ERP feature curve.

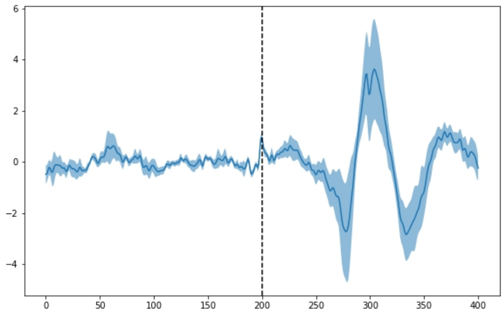

The EEG signal curves of 10, 20, and 40 trials combinations were observed separately, and after more observation screening, we decided to use the curves of 20 trial combinations for the subsequent calculation of the network, because they can use a small number of trial combinations while maintaining the characteristics of the ERP curve.

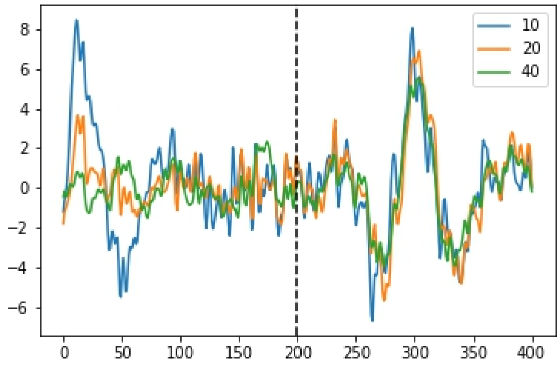

Supplement: Supplementary file 4 [file Presentation_3.PDF]
