# Supplementary material for: Changes in EEG Brain Connectivity Caused by Short-Term BCI Neurofeedback-Rehabilitation Training: A Case Study
Source: Front Hum Neurosci. 2021 Jun 24;15:627100. doi: 10.3389/fnhum.2021.627100 (PMC8336868; doi:10.3389/fnhum.2021.627100)
Supplement: Supplementary file 5 [file Presentation_4.PDF]

The result of three offline(non-feedback) subject:

Mu suppression score

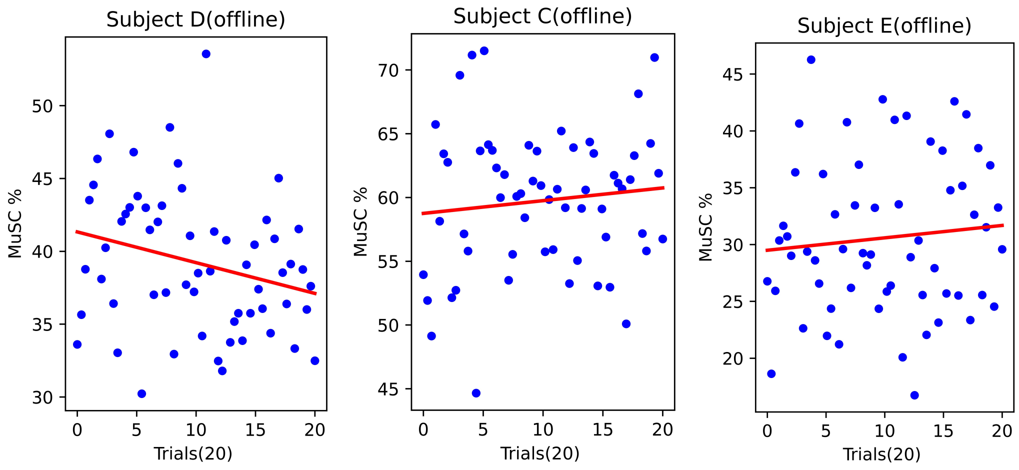

As we can see in the figures, the degree of ERD performance of the three subjects without feedback was unstable and not significantly different from the results of the subjects without feedback in the manuscript.

ERP degree of nodes:

subjectC:

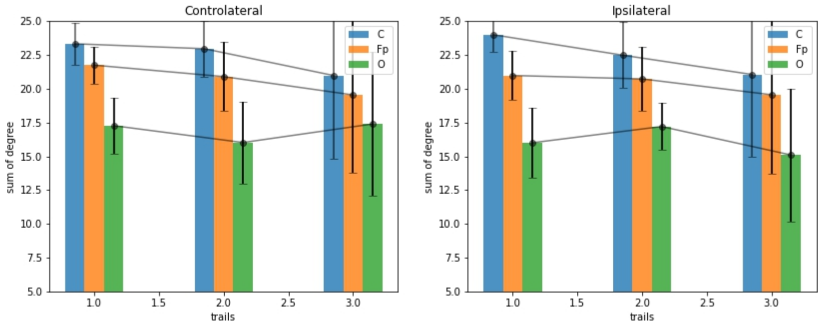

subjectD:

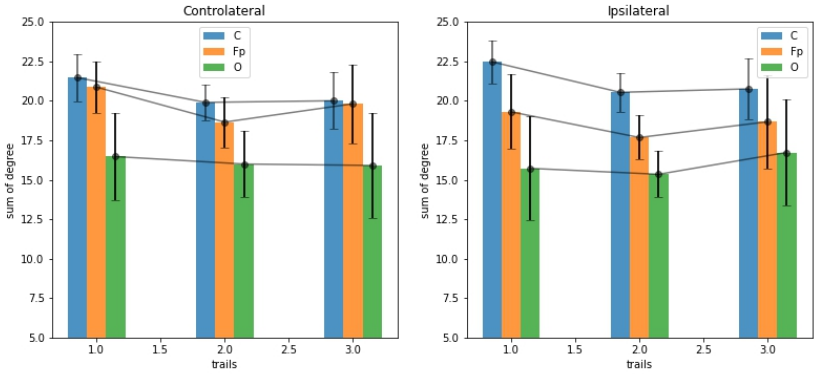

subjectE:

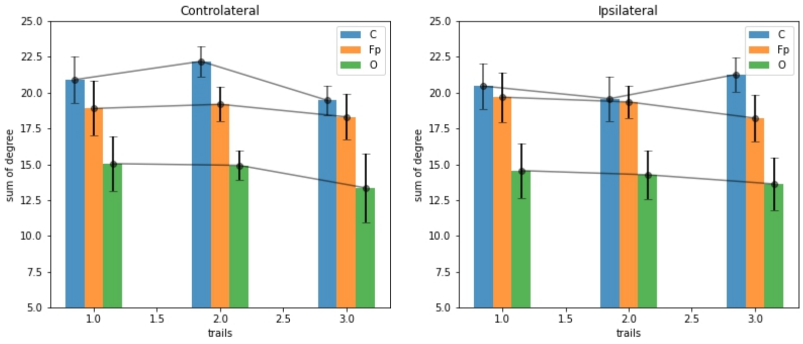

The ERP degree of nodes of the three supplementary subjects without feedback did not differ significantly from the results of the subjects without feedback in the manuscript, neither show a significant decrease compared to the group with feedback.

ERP degree of regions:

subjectC:

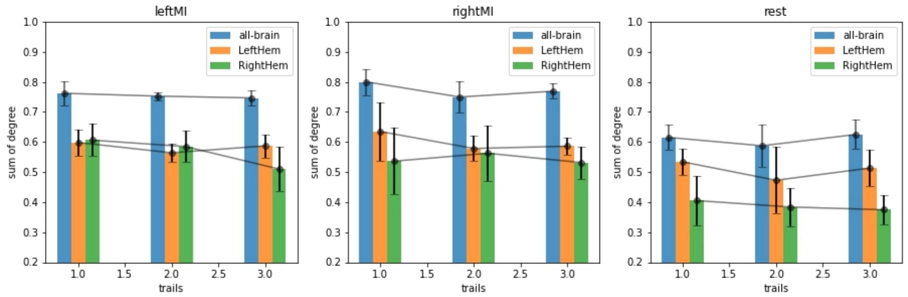

subjectD:

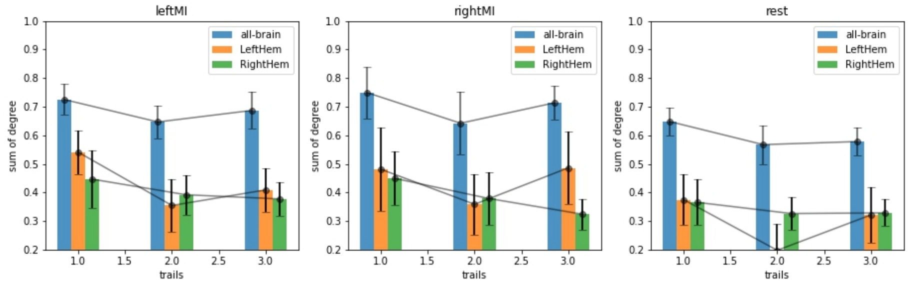

subjectE:

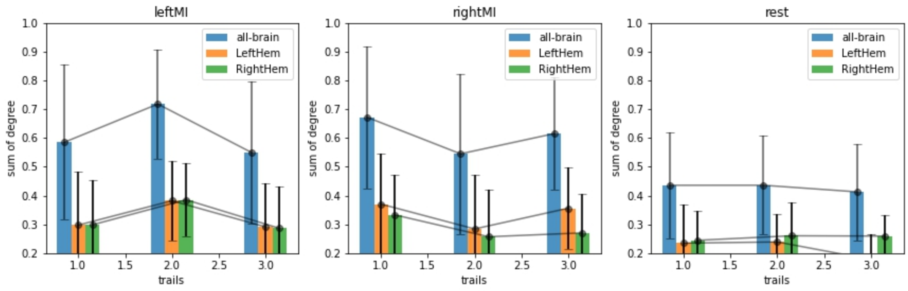

The ERP degree of regions of the three supplementary subjects without feedback did not differ significantly from the results of the subjects without feedback in the manuscript, neither show a significant decrease compared to the group with feedback. The brain connectivity of the task state is significantly higher than that of the rest state, which is also consistent with what is described in the manuscript.
